# Accuracy of Fitbit Wristbands in Measuring Sleep Stage Transitions and the Effect of User-Specific Factors

**DOI:** 10.2196/13384

**Published:** 2019-06-06

**Authors:** Zilu Liang, Mario Alberto Chapa-Martell

**Affiliations:** 1 School of Engineering Kyoto University of Advanced Science Kyoto Japan; 2 Graduate School of Engineering The University of Tokyo Tokyo Japan; 3 Advanced Technology Division CAC Corporation Tokyo Japan

**Keywords:** wearable electronic devices, sleep, validation studies

## Abstract

**Background:**

It has become possible for the new generation of consumer wristbands to classify sleep stages based on multisensory data. Several studies have validated the accuracy of one of the latest models, that is, Fitbit Charge 2, in measuring polysomnographic parameters, including total sleep time, wake time, sleep efficiency (SE), and the ratio of each sleep stage. Nevertheless, its accuracy in measuring sleep stage transitions remains unknown.

**Objective:**

This study aimed to examine the accuracy of Fitbit Charge 2 in measuring transition probabilities among wake, light sleep, deep sleep, and rapid eye movement (REM) sleep under free-living conditions. The secondary goal was to investigate the effect of user-specific factors, including demographic information and sleep pattern on measurement accuracy.

**Methods:**

A Fitbit Charge 2 and a medical device were used concurrently to measure a whole night’s sleep in participants’ homes. Sleep stage transition probabilities were derived from sleep hypnograms. Measurement errors were obtained by comparing the data obtained by Fitbit with those obtained by the medical device. Paired 2-tailed *t* test and Bland-Altman plots were used to examine the agreement of Fitbit to the medical device. Wilcoxon signed–rank test was performed to investigate the effect of user-specific factors.

**Results:**

Sleep data were collected from 23 participants. Sleep stage transition probabilities measured by Fitbit Charge 2 significantly deviated from those measured by the medical device, except for the transition probability from deep sleep to wake, from light sleep to REM sleep, and the probability of staying in REM sleep. Bland-Altman plots demonstrated that systematic bias ranged from 0% to 60%. Fitbit had the tendency of overestimating the probability of staying in a sleep stage while underestimating the probability of transiting to another stage. SE>90% (*P*=.047) was associated with significant increase in measurement error. Pittsburgh sleep quality index (PSQI)<5 and wake after sleep onset (WASO)<30 min could be associated to significantly decreased or increased errors, depending on the outcome sleep metrics.

**Conclusions:**

Our analysis shows that Fitbit Charge 2 underestimated sleep stage transition dynamics compared with the medical device. Device accuracy may be significantly affected by perceived sleep quality (PSQI), WASO, and SE.

## Introduction

### Importance of Consumer Sleep Tracking Devices

Having enough restorative sleep is essential for physical and mental health [1]. In recent years, consumer sleep-monitoring wristbands and associated mobile phone apps have created an effective way for individuals to understand personal sleep patterns or improve sleep quality in daily settings [2]. These devices are relatively affordable, easy to use, and ready to purchase in the consumer market. Most of the consumer wristbands rely on a similar mechanism of clinical actigraphy that infers wake and sleep cycles from limb movement [2]. Newly launched models also incorporate other streams of biosignals, such as heart rate to measure sleep stages. Users can visualize a whole night’s sleep hypnogram (the temporal sequence of sleep stages) and the aggregated sleep parameters, such as total sleep time (TST) and the ratio of each sleep stage on a dashboard [3]. There is increasing evidence that consumer sleep-monitoring wristbands raise awareness of sleep health and have a positive impact on personal sleep hygiene [4-6], though the long-term impact of these technologies has not been elucidated [7]. In the meantime, researchers and clinicians are increasingly adopting consumer wristbands, such as Fitbit devices, as outcome measurement tools in research studies [6,8-14]. Compared with traditional polysomnography (PSG), Fitbit devices significantly reduce the time and monetary cost for longitudinal sleep data collection, and they could provide rich information that was not possible to collect outside sleep laboratories or clinics in the past. Participants can use the devices under free-living conditions, without the need of constant technical support. The new generation of Fitbit devices could also possibly outperform clinical actigraphy, as they leverage multiple streams of biosignals for sleep staging, whereas actigraphy is only able to detect wake and sleep on the basis of limb movement [15].

### Accuracy of Consumer Sleep Tracking Devices

As consumer sleep-monitoring wristbands continue to gain popularity, their limitation in measurement accuracy raised wide concerns on the quality of data collected using these devices [7,16,17]. Data of low quality may mislead users to arrive at wrong conclusions of their sleep. In addition, data quality is of top priority for researchers who intend to use these devices in scientific studies. Therefore, understanding the validity of consumer sleep trackers has practical benefit for both individual users and for the research community. In response to this need, many studies have examined the accuracy of popular sleep trackers compared with medical devices in terms of aggregated sleep metrics, including TST, wake after sleep onset (WASO), sleep efficiency (SE), and sleep stages, that is, light sleep, deep sleep, and rapid eye movement (REM) sleep [18-24]. These studies show that the previous models of consumer wristbands have a common problem of overestimating sleep and underestimating wake [18-20]. Recent models, such as Fitbit Charge 2, that rely on multistreams of biosignals have satisfying performance in measuring TST and SE but fail to produce accurate results in classifying sleep stages [21,24].

**Figure 1:**
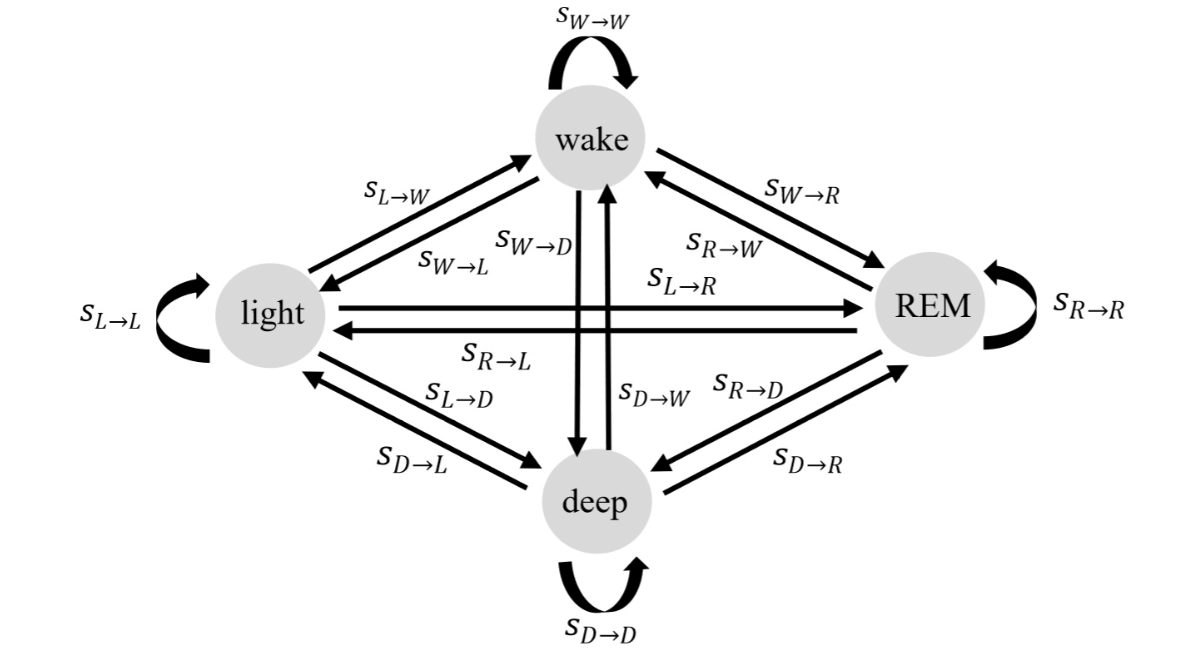
Sleep stage transition dynamics. The W, L, D, R in the subscripts denotes the abbreviation of wake, light sleep, deep sleep, and rapid eye movement sleep.

Although the main body of validation studies has been dominantly focused on polysomnographic metrics (eg, TST, WASO, sensitivity, and specificity) [2,13,24-27], the performance of consumer wristbands in measuring sleep stage transitions remains unknown. Sleep research has shown that sleep stage transition probabilities comprise rich information of sleep patterns, which have been considered more effective than polysomnographic parameters in characterizing sleep stability [28-37]. Sleep stage transition abnormality is an important indicator of sleep disorders [28,32,33,38-43]. Some studies also relied on sleep stage transition probabilities to assess the effect of treatment [44]. The clinical significance of sleep stage transition dynamics suggests the necessity of including relevant metrics (sleep stage transition probabilities) as outcome sleep parameters in validation studies. In Figure 1, a visualization of sleep stage transition dynamics is presented. The total transition probability from a single state to other states (including staying in the same state) is always 1. The *s*_X__→__Y_ represents the transition probability from sleep stage *X* to *Y*. The { *X*, *Y* } are derived from { *W*, *L*, *D*, *R* }, which are abbreviations for wake, light sleep, deep sleep, and REM sleep. For example, *s*_W→R_ denotes the transition probability from wake to REM sleep, and *s*_W→W_ denotes the probability of staying in wake.

### Significance of This Study

This study aimed to examine whether it would accurately measure sleep stage transitions (the transition probabilities among waking, light, deep, and REM sleep) using Fitbit Charge 2. Despite the abundant validation studies, the accuracy of consumer wristbands in measuring sleep stage transition has not been investigated. We also examined the factors that are associated with the measurement errors on sleep stage transition probabilities. Previous validation studies on other types of wearable devices found that device accuracy could vary as a function of the underlying sleep patterns, the population studied, and even how the measurand was defined [45-48]. Along the same line, we selected a set of independent variables (possible predictors), including demographic characteristics of participants, subjective sleep quality measured by Pittsburgh Sleep Quality Index (PSQI) [49], and objective sleep quality derived from medical data. The dependent variables were the absolute percent errors of Fitbit Charge 2 on sleep stage transition probabilities compared with the medical device. The outcomes of this study complement previous validation studies and contribute to the establishment of a holistic view of the capacity of consumer wristbands in measuring sleep structure under free-living conditions. This study also establishes a preliminary reference for researchers who intend to use Fitbit to measure sleep stage transitions and for individual users who rely on Fitbit sleep data to make health decisions.

## Methods

### Recruitment

We recruited participants by distributing posters around the campus of The University of Tokyo. In total, 38 people registered interest through a Web-based form, of whom 28 (74%) were eligible to participate in the study. The inclusion criteria required that the participants were adults (age>18 years), were free of diagnosed chronic conditions, and were able to attend a briefing before the data collection phase. This research was approved by the ethical committee of the University of Tokyo. All participants provided informed consent.

### Study Procedures

A face-to-face briefing was held with each participant individually before the data collection phase. In this meeting, we installed the Fitbit app on participants’ mobile phones and provided verbal instructions on how to use the devices and how to synchronize the Fitbit device with its mobile phone app. Participants were provided with the following items for data collection: a Fitbit Charge 2, a medical device named Sleep Scope, electrodes, chargers, and manuals. At the end of the briefing, participants were asked to fill in a PSQI questionnaire [49] to measure their perceived sleep quality. The PSQI is a widely used instrument for assessing subjective sleep quality averaged over the past 1 month, and a PSQI≥5 is indicative of perceived poor sleep. We collected the PSQI, as it may associate to the measurement accuracy of Fitbit. More details on potential association factors of measurement accuracy will be provided in the next section.

After the briefing, participants measured their sleep using both devices for 3 consecutive nights in their homes to ensure that Fitbit Charge 2 was evaluated in an ecologically valid setting. They were asked to wear the Fitbit on the nondominant wrist during data collection. All participants received a monetary reward when they returned the devices after data collection.

### Data Collection

In this study, we collected sleep data concurrently using Fitbit Charge 2 and a medical device. Fitbit Charge 2 (Fitbit Inc) is a wearable activity wristband with an embedded triaxial accelerometer. It estimates sleep stages for each 30 second period by integrating a user’s movement and heart rate data. With advances in software and hardware, Fitbit Charge 2 has overcome some problems of previous models, and it is able to measure TST and SE with good accuracy [21,24]. A medical sleep monitor named Sleep Scope (Sleep Well Co) was used to obtain the ground truth on sleep hypnograms. Sleep Scope is a clinical-grade single-channel electroencephalogram (Japanese Medical Device Certification 225ADBZX00020000), which was validated against PSG (agreement=86.9%, average Cohen Kappa value =0.75) [50,51]. Sleep Scope was chosen over PSG as it enabled data collection in participants’ homes rather than in a sleep laboratory. This ensures that Fitbit Charge 2 was evaluated in an ecologically valid setting; this also ensures minimalizing the possible disruption of sleep by unfamiliar environment.

In the data collection phase, participants tracked their sleep for 3 consecutive nights in their homes. Following the common practice in sleep science, we analyzed the second night for each participant to remove *the first night effect* [52,53]. If the data of the second night were not valid, then the data of the third night were analyzed. The data of the first night were only selected when neither the second night nor the third night was valid.

Fitbit sleep data were retrieved through the application program interface (API) of Fitbit. Fitbit Charge 2 provides sleep data at 2 levels through public API. The *stage level* data comprise sleep stage levels, including wake, light sleep, deep sleep, and REM sleep. These data are aggregated at 30-second granularity, which complies with the standard sleep staging in the clinical setting. If the *stage level* data are not available, the *classic level* data will be provided as an alternative. *Classic level* data comprise sleep pattern levels, including asleep, restless, and awake, and they are aggregated at a coarser granularity of 60 seconds. In this study, we were interested in the *stage level* sleep data, and the *classic level* data were discarded, as they contained no information on deep sleep, light sleep, and REM sleep.

The data of the medical device were analyzed by the Sleep Well Company, using proprietary automatic scoring algorithms, followed by epoch-by-epoch visual inspection by specialists on the basis of established standards [54], and corrections were added if needed. Fitbit data and medical data were synchronized to make sure that the start time was aligned.

To examine the effect of user-specific factors on measurement accuracy, we also collected data on the factors listed in Table 1. Age and sex were based on self-report, and PSQI was measured by the PSQI questionnaire [49]. Sleep quality metrics were all derived from the medical data.

**Table 1:** A full list of user-specific factors.

Factors	Data type	Data collection method	Cut-off threshold
Age (years)	Ordinal	Self-reported	25
Sex	Nominal	Self-reported	Female or male
PSQI^a^	Ordinal	PSQI questionnaire	5
TST^b^ (min)	Continuous	Sleep scope (medical device)	360
WASO^c^ (min)	Continuous	Sleep scope	30
SOL^d^ (min)	Continuous	Sleep scope	30
SE^e^, %	Continuous	Sleep scope	90.0
Light sleep, %	Continuous	Sleep scope	65.0
SWS^f^, %	Continuous	Sleep scope	20.0
REM^g^, %	Continuous	Sleep scope	20.0
*T*_avg_^h^ (min)	Continuous	Sleep scope	90

^a^PSQI: Pittsburgh Sleep Quality Index.

^b^TST: total sleep time.

^c^WASO: wake after sleep onset.

^d^SOL: sleep onset latency.

^e^SE: sleep efficiency.

^f^SWS: slow wave sleep.

^g^REM: rapid eye movement sleep.

^h^T_avg_: average sleep cycle.

### Statistical Analysis

The overall goal of the analysis was two-fold. We aimed to examine the accuracy of Fitbit Charge 2 in measuring sleep stage transitions compared with a medical device. We were also interested in the associations of user-specific factors with the measurement accuracy of Fitbit Charge 2. All statistical significance levels reported were 2 sided, and statistical analysis was performed using R statistical software version 3.5.3 (The R Foundation)[55].

**Figure 2:**
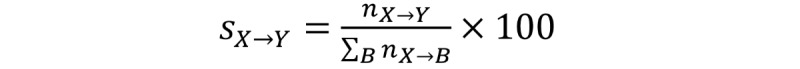
The calculation of sleep stage transition probabilities.

First, descriptive statics of sleep parameters were derived from the medical data. Paired 2-tailed *t* test was used to probe if there were statistically significant differences on sleep patterns between men and women, as well as between participants below 25 years of age and above 25 years of age. Second, sleep stage transition probabilities were calculated by dividing the number of transitions from a specific sleep state to a specific sleep state by the total number of transitions from that specific state to all sleep states (including staying in the same state). As shown in Figure 2, { *X*, *Y*, and *B* } are derived from { *W*, *L*, *D*, and *R* } and *n*_X→Y_ is the number of transitions from sleep stage *X* to *Y* during a whole night’s sleep. The *W*, *L*, *D*, and *R* are the abbreviations for wake, light sleep, deep sleep, and REM sleep. Sleep stage transition probabilities were calculated from Fitbit data and medical data for each participant and then averaged over the whole cohort to obtain the average sleep stage transition probabilities. Systematic difference between the 2 devices was assessed by applying paired *t* test on the sleep stage transition probabilities. A *P* value below .05 was considered statistically significant. The level of agreement between 2 devices was examined using the Bland-Altman plots [56].

**Figure 3:**
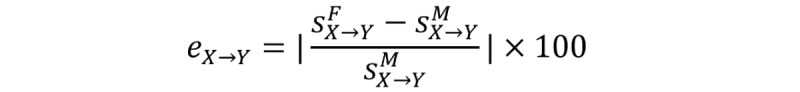
The calculation of absolute percent error.

The absolute percent error *e*_X→Y_ was calculated using the equation in Figure 3, where { *X*, *Y*, and *B* } are derived from { *W*, *L*, *D*, and *R* }, *s*^F^_X→Y_ and *s*^M^_X→Y_ are the transition probability from sleep stage *X* to *Y*, derived from Fitbit data and medical data.

To examine the effect of user-specific factors on absolute percent error, the dataset was divided into 2 subsets according to the cut-off threshold values listed in Table 1. Wilcoxon signed–rank test was conducted to examine if there were significant differences between the 2 subsets in terms of the outcome sleep metrics (sleep stage transition probabilities). The selection of cut-off threshold values was in line with literature in sleep science [49,57].

## Results

### Descriptive Statistics

A total of 28 young adults without chronic diseases participated in the study. A total of 5 participants were excluded from analysis because of failure to obtain *stage level* sleep data with Fitbit. That is, only *classic level* sleep data were obtained from these participants; the data had no information on light, deep, and REM sleep. Therefore, it was not possible to calculate sleep stage transition probabilities for these participants. The final dataset thus comprises sleep data from 23 participants (men:women=14:9). This number of participants is comparable with other validation studies [20,27,58-61]. All the participants were university students between 21 to 30 years old (mean 24.3, SD 2.7). A total of 8 out of the 23 participants had a PSQI higher than 5, which was indicative of unsatisfied sleep quality. Statistically significant differences were found between men and women in terms of wake time (women: 9.7 min; men: 22.8 min; *P*=.02) and the ratio of sleep stage 1 (women: 7.7(%); men: 14.3(%); *P*=.02). We also compared the sleep patterns between participants below and above 25 years. Statistically significant differences were found in terms of TST (below 25 years: 308.7 min; above 25 years: 396.8 min; *P*=.03), transition probability from deep sleep to light sleep (below 25 years: 5.5%; above 25 years: 1.5%; *P*=.02), and the probability of staying in light sleep (below 25 years: 85.3(%); above 25 years: 94.8(%); *P*=.008).

### Systematic Differences

Table 2 presents the estimated sleep stage transition probabilities derived from medical data and Fitbit data, as well as the results of paired *t* test. We calculated sleep stage transition probabilities individually for each participant and then averaged results across the whole cohort. It is shown that the following transitions rarely occurred: deep sleep to REM sleep and wake, light sleep to REM sleep, REM sleep to deep sleep, and REM sleep to light sleep. The *t* test results indicated that there were significant differences between the sleep stage transition probabilities measured by Fitbit and those measured by the medical device. Fitbit deviated from the medical device on all the transition probabilities except for the transition probability from light sleep to REM sleep (*s*^F^_L→R_ = 0.9%; *s*^M^_L→R_ =1.7%), the transition probability from deep sleep to wake (*s*^F^_D→W_ = *s*^M^_D→W_ =0.2%), and the probability of staying in REM sleep stage (*s*^F^_R→R_ = *s*^M^_R→R_ =96.9%). In general, Fitbit underestimated sleep stage transition dynamics. The probabilities of staying in a specific sleep stage were significantly overestimated, whereas the probabilities of transitions from a specific stage to a different stage were mostly underestimated.

**Table 2:** Average sleep stage transition probabilities (%) and results of paired *t* test. Data are displayed as mean and ±95% CI.

Sleep stage		Wake	Light	Deep	REM^a^
**Wake**					
	Medical	53.7 (44.0-63.3)	43.6 (33.8-53.4)	0.2 (0.0-0.4)	2.6 (1.5-3.7)
	Fitbit	89.8 (81.2-98.3)	5.5 (4.3-6.7)	0.2 (0.0-0.5)	0.2 (0.0-0.5)
	*P* value	<.001	<.001	.83	<.001
**Light**					
	Medical	2.6 (2.0-3.3)	92.6 (90.9-94.4)	3.9 (2.1-5.8)	0.8 (0.7-0.9)
	Fitbit	0.5 (0.3, 0.6)	97.8 (97.6-98.1)	1.1 (0.9-1.3)	0.5 (0.3-0.7)
	*P* value	<.001	<.001	.005	.02
**Deep**					
	Medical	2.5 (0.7-4.3)	57.7 (43.8-71.6)	35.5 (22.6-48.4)	0.0 (0.0-0.0)
	Fitbit	0.2 (0-1.8)	3.8 (2.9-4.6)	94.9 (93.4-96.4)	1.1 (0.4-1.8)
	*P* value	.02	<.001	<.001	.002
**REM**					
	Medical	2.0 (1.6-2.4)	0.9 (0.7-1.2)	0.0 (0.0-0.0)	96.9 (96.5-97.5)
	Fitbit	0.1 (0.0-0.2)	1.7 (0.7-2.6)	1.2 (0.3-2.2)	96.9 (96.0-98.0)
	*P* value	<.001	.14	.01	>.99

^a^REM: rapid eye movement.

### Level of Agreement and Correlations

**Figure 4:**
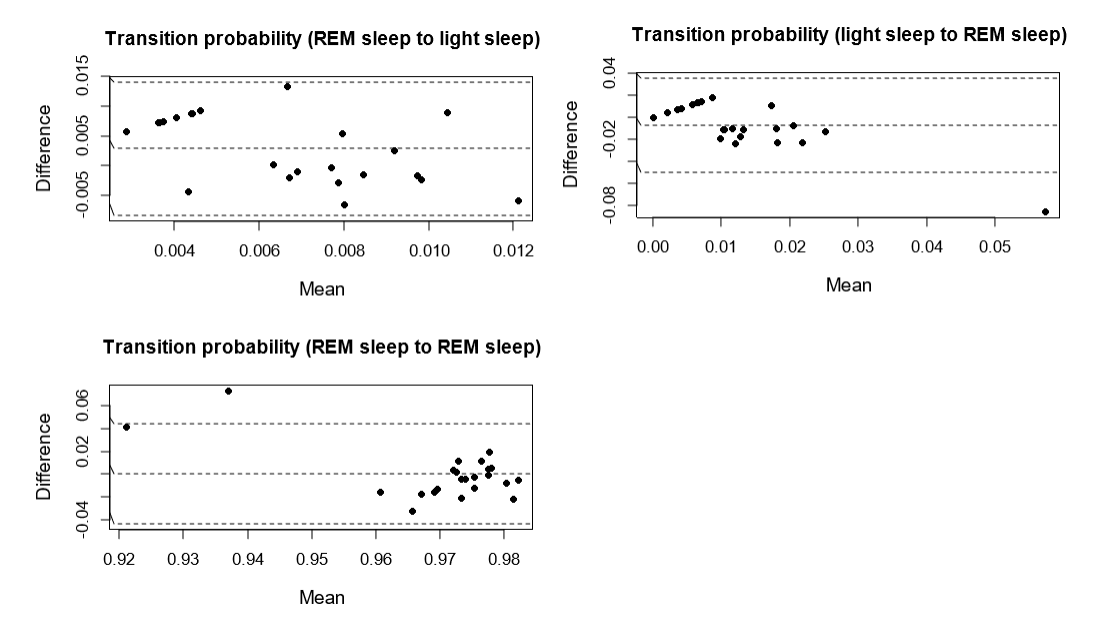
Bland-Altman plots assessing the level and limits of agreement between Fitbit Charge 2 and medical device on the transition probabilities from rapid eye movement (REM) sleep to light sleep, from light sleep to REM sleep, and the probability of staying in REM sleep. The dashed line in the middle represents the mean difference, whereas the upper and lower dashed lines represent the upper limit of agreement and the lower limit of agreement.

**Figure 5:**
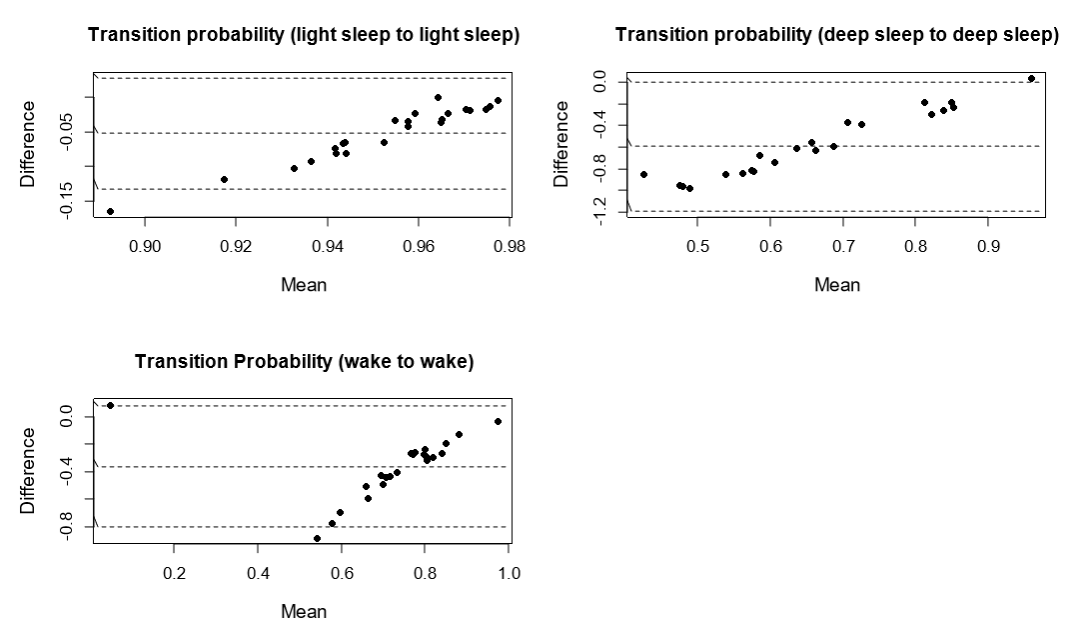
Bland-Altman plots assessing the level and limits of agreement between Fitbit Charge 2 and medical device on the probability of staying in light sleep, in deep sleep, and in wake.

**Figure 6:**
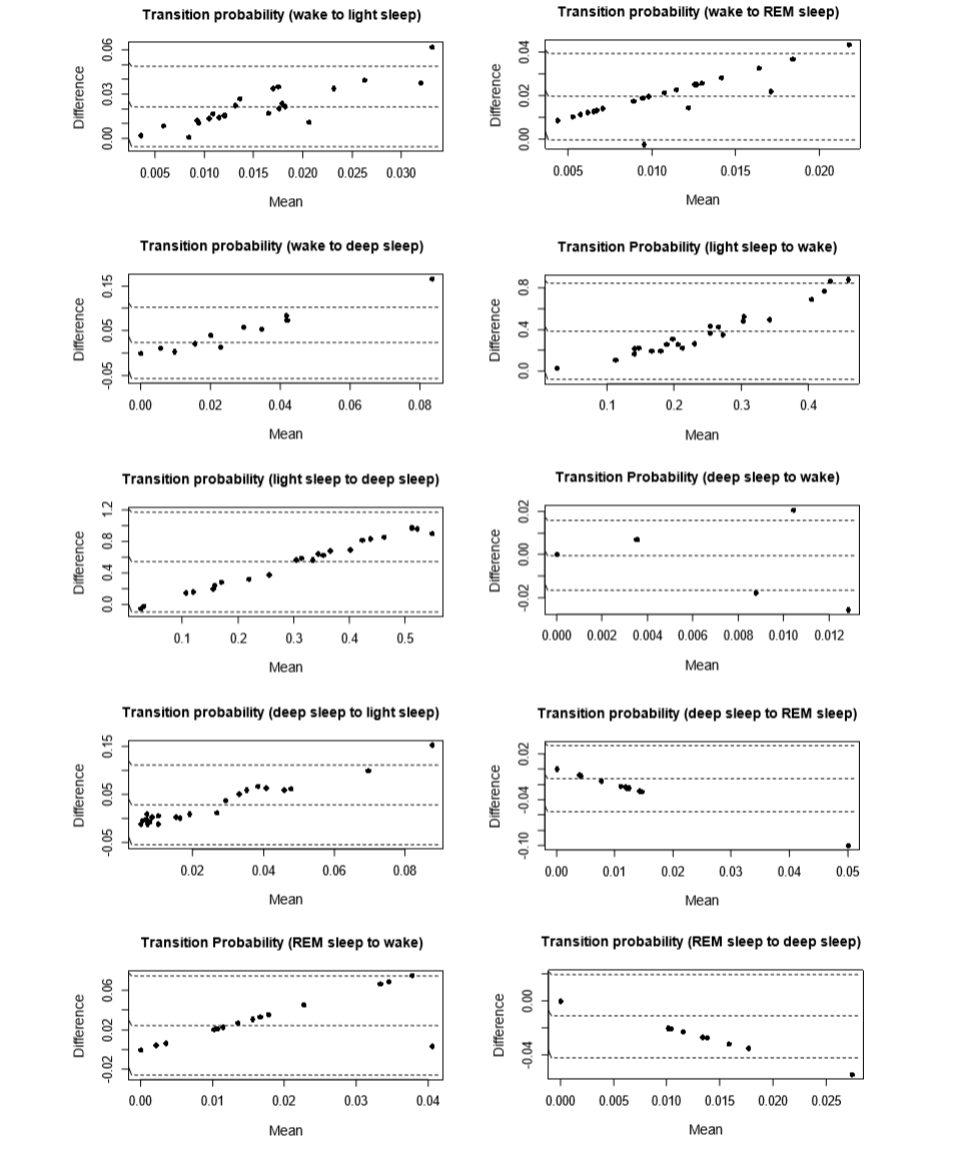
Bland-Altman plots assessing the level and limits of agreement between Fitbit Charge 2 and medical device on the transition probabilities from wake to light sleep, from wake to rapid eye movement (REM) sleep, from wake to deep sleep, from light sleep to wake, from light sleep to deep sleep, from deep sleep to wake, from deep sleep to light sleep, from deep sleep to REM sleep, from REM sleep to wake, and from REM sleep to deep sleep.

Figures 4-6 show the Bland-Altman plots comparing Fitbit Charge 2 with the medical device. Device discrepancies for sleep outcomes are plotted as a function of the medical outcomes for each individual. The mean bias ranged from 0% (*s*_R→R_ and *s*_D→W_) to approximately 60% (*s*_L→D_). No more than 2 participants were situated outside the lower limit of agreement or the upper limit of agreement.

In line with previous studies [62,63], we defined the acceptable error range as e_i_ ≤5%, as this approximates a widely acceptable standard for statistical significance in literature [64]. On the basis of this criterion, no systematic bias was found between Fitbit and the medical device in measuring *s*_W→L_, *s*_W→R_, *s*_L→R_, *s*_D→W_, *s*_R→L_, *s*_R→D_, and *s*_R→R_.

Figure 4 shows that no trend was found between the difference and the mean of *s*_R→L_, *s*_L→R_ and *s*_R→R_. In contrast, Figure 5 and Figure 6 show clear trends that the measurement differences were greater for lower *s*_L→L_, *s*_D→D_, and *s*_W→W_, and the differences were greater for higher *s*_W→L,_ *s*_W→R_, *s*_W→D_, *s*_L→W_, *s*_L→D_, *s*_D→W_, *s*_D→L_, *s*_D→R_, *s*_R→W_, and *s*_R→D_. These findings suggest that the accuracy of Fitbit Charge 2 in measuring sleep stage transitions could be deteriorated as sleep became more dynamic (more transitions between different sleep stages).

### Effect of User-Specific Factors

The results of Wilcoxon signed–rank test showed that good subjective sleep quality indicated by PSQI as lower than 5 was associated with decreased errors in the probability of staying in deep sleep stage (PSQI<5, 132.1±173.1%; PSQI≥5, 346.8±250.0%; *P*=.04), but it was associated with increased errors in transition probability from waking to REM sleep (PSQI<5, 100.0±0.0%; PSQI≥5, 85.1±25.5%; *P*=.02).

Wake time longer than 30 min was associated with increased errors in transition probability from light sleep to REM sleep (WASO≥30, 265.8±176.5; WASO<30, 103.9±49.1%; *P*=.02), but it was associated with decreased errors in transition probability from light sleep to wake (WASO≥30, 78.6±10.2%; WASO<30, 86.7±8.6%; *P*=.049), as well as the probability of staying in wake (WASO≥30, 117.3±269.5%; WASO<30, 125.2±103.6%; *P*=.006).

SE above 90% was associated with increased measurement errors in transition probability from REM sleep to light sleep (SE>90, 107.1±53.2%; SE≤90%, 55.9±40.4%; *P*=.047).

In addition, age below 25 years (age<25, 7.9±5.4%; age≥25, 3.1±2.3%; *P*=.01), sleep onset latency (SOL) shorter than 30 min (SOL<30, 8.6±5.8%; SOL≥30, 4.1±3.4%; *P*=.02), and deep sleep ratio above 20% (slow wave sleep; SWS<20%, 3.9±3.5%; SWS≥20, 9.5±5.2; *P*=.007) were associated with slight increased measurement error in the probability of staying in light sleep stage. Nevertheless, the average errors were no more than 10% in all the corresponding cases.

No significant associations were found between measurement errors of Fitbit and other factors, including sex, TST, SOL, light sleep ratio, REM sleep ratio, and T_avg_.

## Discussion

### Principal Findings

We have demonstrated a numerical comparison on sleep stage transition probabilities between Fitbit Charge 2 and the medical device. The level and limits of agreement between the 2 types of devices were illustrated using Bland-Altman plots. The results of Wilcoxon signed–rank test were presented to demonstrate the associations between user-specific factors and measurement errors. This study generated 2 main findings. First, we found that Fitbit Charge 2 underestimated sleep stage transition dynamics compared with the medical device. Second, device accuracy was mainly associated with 3 user-specific factors: subjective sleep quality measured by PSQI, WASO, and SE.

Sleep stage transition analysis has been used to characterize sleep continuity and the temporal stability of non-REM and REM bouts in sleep science [28-30,32,40,44]. In this study, the sleep stage transition probabilities derived from the medical data demonstrated interesting patterns. As expected, the probability for any sleep stage to stay in the same stage was constantly higher than that for this stage to change to a different stage. Direct transition between deep sleep and REM sleep rarely happened. The probability of transitions from wake to deep sleep or from wake to REM sleep was low. Similarly, the probability of transition from deep sleep to wake was also low. These characteristics were consistent with findings reported in previous sleep studies on sleep stage transition patterns in healthy people [31,44].

Sleep stage transition is the result of complex interactions among many brain regions. Not being able to detect markers in brainwaves, such as k-complexes [54], consumer wristbands have limited performance in classifying sleep stages. Previous studies show that Fitbit Charge 2 devices significantly overestimated light sleep and underestimated deep sleep when validated in lab settings [21], whereas they underestimated deep sleep and overestimated light and REM sleep when validated under free-living conditions [24]. This study complements previous findings and contributes new insights into Fitbit’s capacity in capturing sleep stage transitions. Overall, we observed that Fitbit Charge 2 significantly deviated from the medical device in measuring sleep stage transition dynamics. Notably, the average probabilities of staying in wake stage and deep stage measured by Fitbit were significantly higher than those measured by the medical device. In contrast, Fitbit underestimated the probabilities of stage transitions from light sleep to wake and from light sleep to deep sleep. This is probably because of the misclassification of wake and deep sleep epochs to light sleep [21]. Systematic bias (between 40% and 60%) was illustrated in the Bland-Altman plots on these sleep stage transition probabilities. On the other hand, no systematic bias and mean difference were observed in measuring the probability of staying in REM sleep stage. This result provides complementary evidence to the finding in the study by De Zambotti et al [21] that Fitbit Charge 2 agreed well to medical devices in detecting REM sleep.

A unique aspect of this study is that we also examined the effect of user-specific factors and found multiple associations. Our analysis showed that subjective sleep quality measured by PSQI, wake after WASO, and SE were significantly strong predictors of measurement errors in sleep stage transition probabilities. Age, SOL, and deep sleep ratio were significant but weak predictors, whereas sex, TST, light sleep ratio, REM sleep ratio, and average sleep cycle were not associated with the measurement errors of Fitbit.

Despite the finding from previous validation studies that poor sleep quality is associated with deteriorated performance of sleep monitoring devices in measuring polysomnographic sleep metrics [21,25,65], this study reveals that the relationship is more complicated between sleep quality and device accuracy in measuring sleep stage transitions. Indeed, we found that good subjective sleep quality (PSQI<5) was associated with decreased measurement error in the probability of staying in deep sleep stage, and less fragmented sleep (WASO<30 min) was associated with decreased errors in transition probability from light sleep to REM sleep. Nevertheless, it is also found that good sleep characterized by quick sleep onset (SOL<30 min), high ratio of deep sleep (SWS>20%), good subjective feeling (PSQI<5), short awakenings (WASO<30 min), and high SE (SE>90%) were associated with increased measurement errors in different outcome transition probabilities. This result contradicts previous findings on actigraphy that deteriorated sleep (eg, long WASO and SOL) increased measurement errors [21,25,65]. This disparity suggests that findings related to clinical actigraphy should not be generalized to consumer wristbands without further validation.

In addition, age was found to be a significant but weak predictor of measurement errors. Participants in the age range of 25 to 30 had decreased measurement errors in the probability of staying in light sleep stage compared with those younger than the age of 25. As age has been widely recognized as a significant factor that alters sleep patterns [43,57], the effect of age may also be traced back to the difference in underlying sleep patterns. The medical sleep data showed that younger participants generally had shorter sleep and higher sleep stage transition dynamics (transition from deep sleep to light sleep), which may account for the increase in measurement errors. Nevertheless, this finding should not be generalized to a wide range of age groups because of the restricted sampling of age in this study. Further studies are needed to systematically examine the effect of age on device accuracy.

Our findings complement those of previous validation studies on consumer wristbands for sleep tracking in general. Fitbit Charge 2 has demonstrated satisfying performance in measuring TST and SE, but it remains incapable of classifying sleep stages with good accuracy [21,24]. Our findings show that Fitbit Charge 2 may also underestimate sleep transition dynamics, and it should thus be used with caution. This study establishes a preliminary reference for researchers who intend to use the Fitbit device to measure sleep stage transitions in scientific studies, and this study suggests that both perceived and objective sleep patterns may need to be considered when choosing sleep monitoring tools.

### Limitations

This study is subject to the following limitations. First, the participants represent a young healthy population that was free of sleep disorders or chronic diseases. Therefore, the results cannot be generalized to older or clinical populations. Second, the data collection phase was not longitudinal in nature, and only 1 night of sleep from each participant was analyzed. Thus, the results may fail to count intrapersonal variations. Third, the list of potential affecting factors investigated in this study was not exhaustive, and it may be affected by restricted sampling. Further research should address these limitations by including a diverse population, extending data collection duration, and examining the effect of other potential predictors of device accuracy.

### Conclusions

We have demonstrated that Fitbit Charge 2 significantly underestimated sleep stage transition dynamics compared with the medical device and that measurement accuracy could be mainly affected by perceived sleep quality, sleep continuity, and SE. Despite the positive trend of enhanced accuracy for the latest consumer wearable sleep trackers, the limitation of these devices in detecting sleep stage transition dynamics needs to be recognized. As an outcome measurement tool, Fitbit Charge 2 may not be suited for research studies related to sleep stage transitions or for health care decision making. Further research should focus on enhancing the accuracy of these consumer wristbands in measuring not only polysomnographic parameters but also sleep stage transition dynamics.

## Abbreviations

API: application programming interface
PSG: polysomnography
PSQI: Pittsburgh Sleep Quality Index
REM: rapid eye movement
SE: sleep efficiency
SOL: sleep onset latency
SWS: slow wave sleep
TST: total sleep time
WASO: wake after sleep onset
